# Ionothermal synthesis of magnetic N-doped porous carbon to immobilize Pd nanoparticles as an efficient nanocatalyst for the reduction of nitroaromatic compounds

**DOI:** 10.1038/s41598-023-35998-5

**Published:** 2023-10-16

**Authors:** Sahar Taheri, Majid M. Heravi, Asma Saljooqi

**Affiliations:** 1 Department of Chemistry, Faculty of Physics and Chemistry, Alzahra University, Tehran, Iran; 2 Department of Chemistry, Shahid Bahonar University of Kerman, Kerman, Iran

**Keywords:** Environmental chemistry, Catalysis

## Abstract

Carbon materials play important roles as catalysts or catalyst supports for reduction reactions owing to their high porosity, large specific surface area, great electron conductivity, and excellent chemical stability. In this paper, a mesoporous N-doped carbon substrate (exhibited as N–C) has been synthesized by ionothermal carbonization of glucose in the presence of histidine. The N–C substrate was modified by Fe_3_O_4_ nanoparticles (N–C/Fe_3_O_4_), and then Pd nanoparticles were stabilized on the magnetic substrate to synthesize an eco-friendly Pd catalyst with high efficiency, magnetic, reusability, recoverability, and great stability. To characterize the Pd/Fe_3_O_4_–N–C nanocatalyst, different microscopic and spectroscopic methods such as FT-IR, XRD, SEM/EDX, and TEM were applied. Moreover, Pd/Fe_3_O_4_–N–C showed high catalytic activity in reducing nitroaromatic compounds in water at ambient temperatures when NaBH_4_ was used as a reducing agent. The provided nanocatalyst's great catalytic durability and power can be attributed to the synergetic interaction among well-dispersed Pd nanoparticles and N-doped carbonaceous support.

## Introduction

Amines, particularly aniline and its derivatives, have an enormous market section in the organic chemical industry^1,2^. Aromatic amines are important intermediates in the construction of different chemicals such as polymers, pigments, drugs, chelating agents, and surfactants^3–6^. Reducing nitroaromatics is not only an ordinary and straight way to achieve amines but also one of the most important chemical processes in industries^7,8^. Variant hydrogen donors including H_2_^9^, sodium borohydride^10,11^, formic acid^12^, and N_2_H_4_^13^ have been utilized a lot to hydrogenate nitroaromatics with diverse catalysts^3^. Hence, metal-based heterogeneous and homogeneous catalysts have extensively been applied for reduction reactions of nitroaromatic compounds^14,15^. Till now, different kinds of heterogeneous catalysts utilizing noble metals such as Pd^16–18^, Au, Pt, and Rh, as well as non-noble metal catalysts like Cu, Ni, Fe, and Co, have been reported to the direct reduction reaction of nitroaromatic compounds^19–21^.

Between variant heterogeneous catalysts, supported metal nanocatalysts have gained substantial attention in hydrogenation reactions because of their high catalytic performance, reusability, and stability^22–24^. However, appropriate support is necessary to earn the intended catalytic capability by forming strong interactions with metal nanoparticles^25,26^. Plenty of solid supports, particularly silica, polymers, MOF, metal oxides, and mesoporous carbon, are used for immobilizing nanoparticles. Nevertheless, in the meantime, carbon-based materials have been extensively used as support for many chemical reactions^27,28^. Mesoporous carbons are supreme supports because of their outstanding properties, such as large specific surface areas, excellent chemical stability, porosity, great electron conductivity, and high mechanical strength^29^.

Heteroatom doping is an efficient technique to improve the useful properties and coordinate the electronic states of carbon-based materials. As an example, this strategy leads to improved catalytic stability and modification of geometric and electronic structures of carbon materials^30,31^. Embedment of heteroatoms such as B, P, S, and N into the carbon materials framework introduces multiple active sites for immobilizing nanoparticles^32^. Among these heteroatoms, N is the rational option for carbon-based materials, as the atomic sizes of C and N are alike. N-doped porous carbons can be prepared by utilizing diverse N-rich precursors like NH_3_^33^, melamine^34^, pyridine^35^, urea^36^, thiourea^37^, cyanamide^38^, L-lysine, tyrosine, and L-cysteine^39^. In addition, N-doped porous carbon substrates are prepared and synthesized by various methods, including hard and soft templates^40^, thermal polymerization^41^, post-treatment^42^, and chemical vapor deposition (CVD)^43^, solvothermal and ionothermal methods^44,45^.

The synthesis of N-doped porous carbons by the ionothermal method proceeds from the suggested procedures by Morris and co-workers in 2004. They synthesized new classes of zeolites utilizing eutectic compounds or ionic liquids as structure-directing agents and solvents^46,47^. The preparation of carbonaceous materials such as N-doped carbons following the principles of ionothermal has been reported in the literature as ionothermal and molten-salt synthesis processes^48,49^. In further studies in ionothermal approaches, the use of different kinds of salt like ZnCl_2_, LiCl, KCl, LiBr, and KBr as reaction-directing agents became common and admitted as a hard template^50–52^. The ionothermal method makes it possible to synthesize porous carbons with a super high surface area, hierarchical structure, and high heteroatom doping^53,54^.

In this paper, we used the good solvability of zinc chloride in water (green solvent) and developed a simple ‘‘wet chemistry’’ ionothermal method to prepare N-doped porous carbon substrate with high N-rich content (N > 6 wt%). Also, we utilized glucose and histidine as inexpensive and sustainable carbon and nitrogen precursors. Finally, Pd nanoparticles were synthesized and immobilized on the magnetic N-doped porous carbon substrate. The Pd/Fe_3_O_4_–N–C nanocatalyst has been synthesized, and the catalytic performance has also been studied in reducing different aromatic compounds bearing the nitro group.

## Experimental

### Materials

Zinc Chloride (ZnCl2), glucose (C_6_H_12_O_6_), histidine (C_6_H_9_N_3_O_2_), palladium (II) chloride (PdCl_2_, 99%), ammonium hydroxide solution (NH_4_OH, 25%), ferrous chloride tetrahydrate (FeCl_2_·4H_2_O, 98%), ferric chloride hexahydrate (FeCl_3_·6H_2_O, 97%), acetonitrile (CH_3_CN) and hydrazine hydrate (N_2_H_4_.H_2_O, 80% solution in water) were all procurement from Merck and Sigma-Aldrich.

### Synthesis of Pd/Fe_3_O_4_–N–C nanocatalyst

#### Synthesis of nitrogen-doped carbon substrate (N–C substrate)

To prepare a nitrogen-doped carbon substrate, in the first step, glucose (33.3 mmol), zinc chloride (66.03 mmol), and histidine (20 mmol) were blended well in a mortar to procure a homogeneous composition. Subsequently, 20 mL of water was added to the mixture and was mixed well. Next, the resulting combination was decanted into a 50 mL-Teflon autoclave and was placed in an oven at 180 °C for 20 h. After cooling to the ambient temperature, the admixture was rinsed with distilled water multiple times and immersed in hydrogen chloride (0.25 M) overnight. Subsequently, the black residue was washed with ethanol and water to eliminate the excess salt and hydrogen chloride. Eventually, the resulting product was collected by centrifuge and dried at 40 °C.

#### Synthesis of the magnetic substrate (N–C/Fe_3_O_4_)

Briefly, 0.5 g of the obtained sample (N–C substrate) was dispersed in distilled water (120 mL). A blend of 2.5 mmol of FeCl_2_.4H_2_O and 5 mmol of FeCl_3_.6H_2_O were added and stirred for 1 h at room temperature. Then under reflux conditions, the reaction temperature was brought to 60 °C while 10 mL of NH_4_OH was added dropwise to the above mixture and was continuously stirred for another 1 h. Later, the magnetic sediment was separated by an external magnet, washed three times with distilled water, and dried up at room temperature.

#### Synthesis of Pd nanoparticles on the magnetic substrate (Pd/Fe_3_O_4_–N–C)

**Figure 1:**
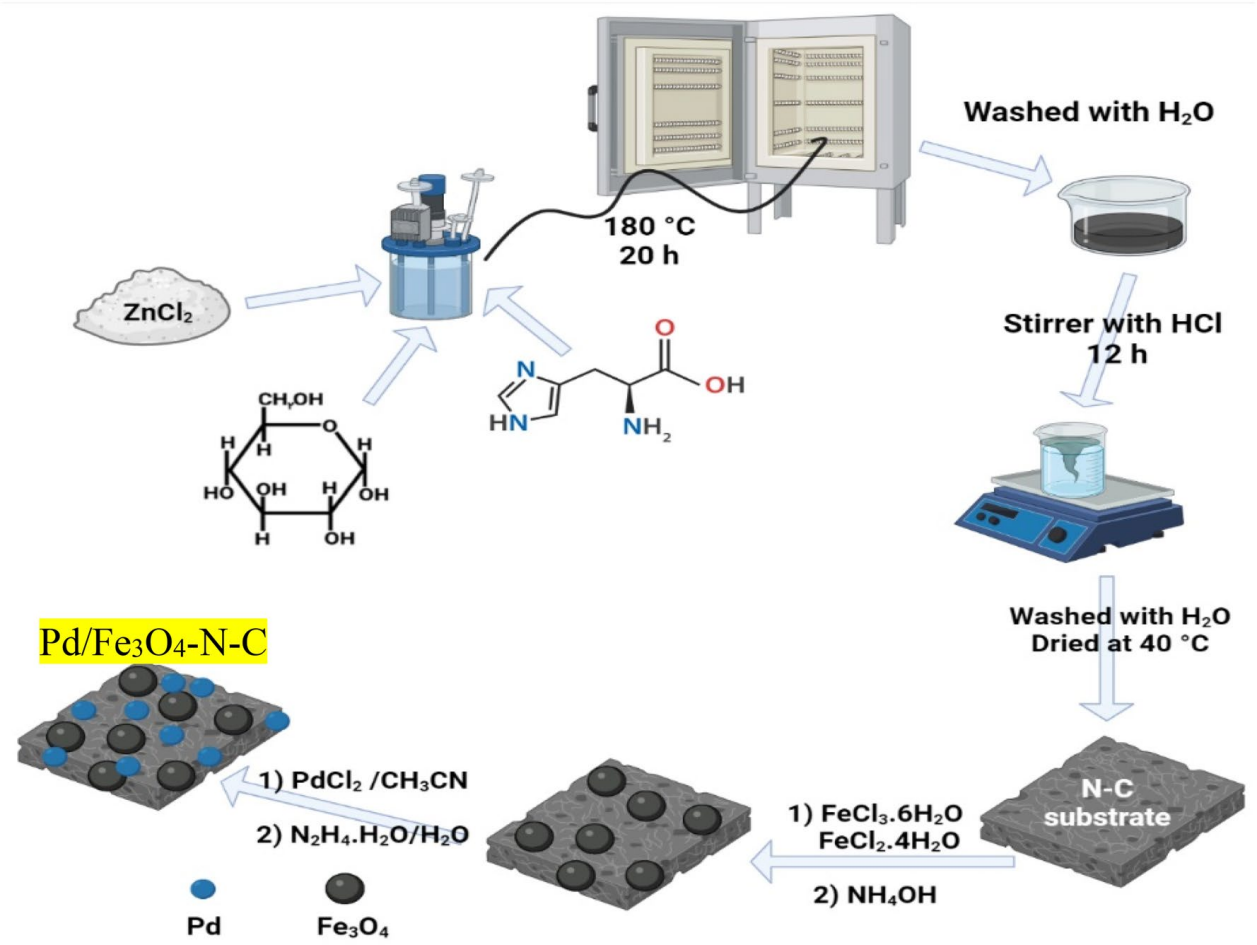
The process of Pd/Fe_3_O_4_–N–C nanocatalyst synthesis.

In the final step to synthesize Pd nanoparticles on the magnetic nitrogen-doped carbon substrate, 0.2 mmol palladium (II) chloride, and 60 mL acetonitrile were initially constantly stirred at 55 °C for 1 h. Afterward, 250 mg of the previous step precipitate was added to the above solution and stirred at the same temperature for another 30 min, and then 0.5 mL of a solution of hydrazine hydrate (0.5 mL) in deionized water (2 mL) was added dropwise and stirred for 24 h. The final catalyst was magnetically separated, deterged multiple times with distilled water, and dried at room temperature. The procedure of Pd/Fe_3_O_4_–N–C nanocatalyst synthesis is demonstrated in Fig. 1.

### Performances of Pd/Fe_3_O_4_–N–C nanocatalyst in the hydrogenation reaction

The prepared Pd/Fe_3_O_4_–N–C nanocatalyst by this ion-thermal method had high catalytic efficiency toward the hydrogenation of nitroaromatics. In an aqueous solution, reduction reactions were conducted at ambient temperature, with NaBH_4_ as the reducing agent. Accordingly, 3 mL of water and 0.5 mmol of nitroaromatic compounds in a round-bottom flask (10 mL) were stirred potently at room temperature. Afterward, 5 mg of Pd/Fe_3_O_4_–N–C and three mmol sodium tetrahydroborate were added to the mixture and stirred as long as the reaction was complete. Thin-layer chromatography was used to monitor the progress of the reduction reaction (n-hexane: ethyl acetate 7:3). At the end of the reaction, Pd/Fe_3_O_4_–N–C nanocatalyst was removed using an external magnet, washed out with distilled water and ethanol, and dried to reuse for the next cycle. Additionally, the final product was recrystallized for purification.

## Results and discussion

### Characterization of Pd/Fe_3_O_4_–N–C nanocatalyst

#### FT-IR Spectroscopy of Pd/Fe_3_O_4_–N–C

**Figure 2:**
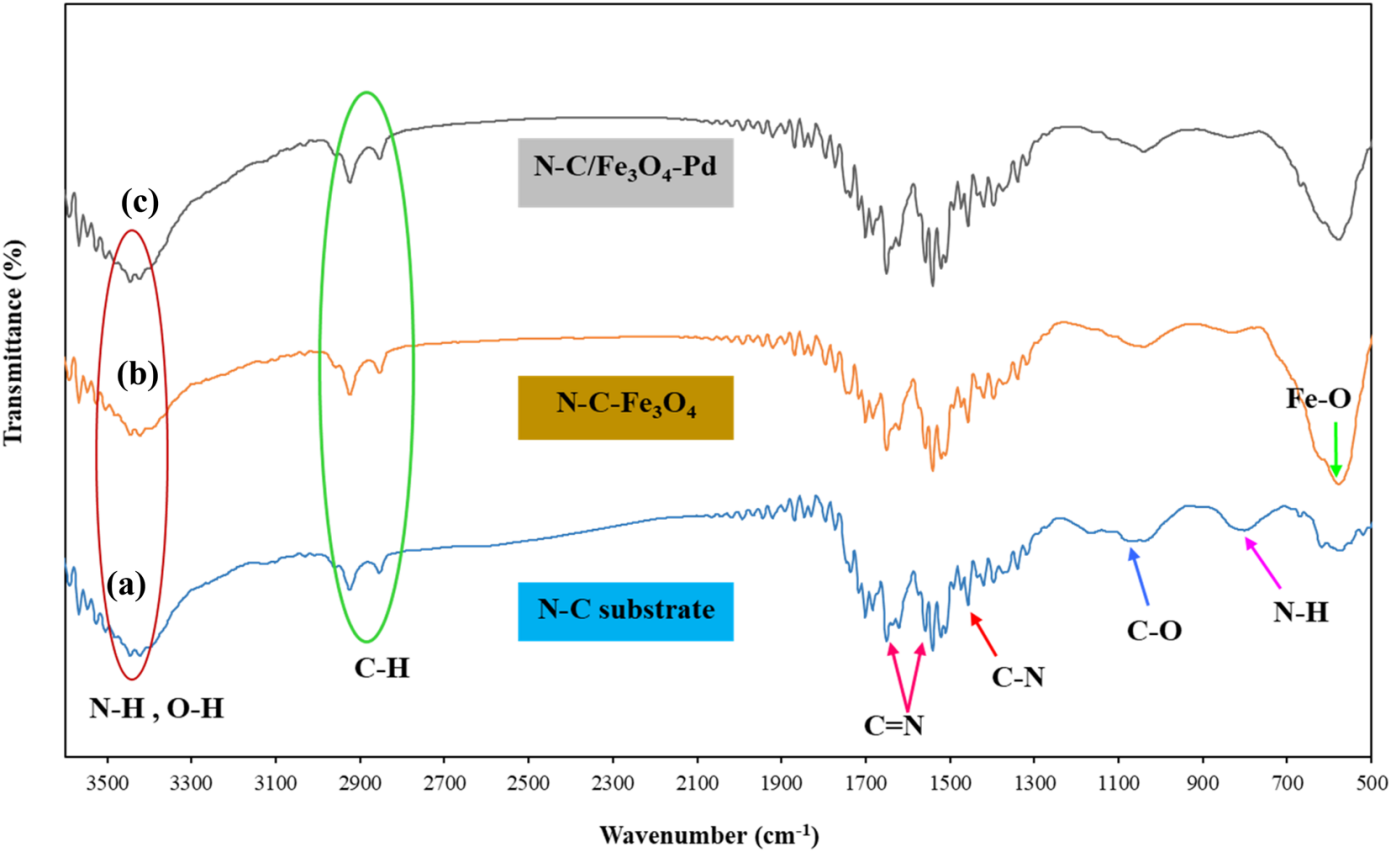
FT-IR spectra of (**a**) N–C substrate, (**b**) N–C/Fe_3_O_4_, and (**c**) Pd/Fe_3_O_4_–N–C.

For a more detailed peruse of the structure of the Pd/Fe_3_O_4_–N–C nanocatalyst, the FT-IR spectrum of its construction steps of (a) N–C substrate, (b) N–C/Fe_3_O_4_ (c) Pd/Fe_3_O_4_––N–C were investigated which is shown in Fig. 2. As shown in Fig. 2a, the absorption bands in 814 cm^−1^ and 1065 cm^−1^ are related to the N–H bending vibration and the C–O stretching vibration, respectively. The peak occurring in the 1434 cm^−1^ mainly belongs to the stretching vibrations of the C–N bond. The two absorption bands observed in 1559 cm^−1^ and 1621 cm^−1^ correspond to the stretching vibration of the C=N bond. The absorption bands at 2854 cm^−1^ and 2924 cm^−1^ indicate the stretching vibration of the C-H bond. The broad peak at 3400 cm^−1^ refers to the stretching vibration of N–H and O–H. Therefore, these outcomes reveal the presence of nitrogen in the carbonaceous framework. In Fig. 2b, the band that developed at 577 cm^−1^ characterized vibration of the Fe–O bond, representing the Fe_3_O_4_ formation. In Fig. 2c, it is observed that by adding palladium nanoparticles to the surface of the magnetic substrate, no significant change has been made, which indicates that the N–C/Fe_3_O_4_ substrate was stable during the synthesis of palladium nanoparticles^55^.

#### X-ray diffraction of Pd/Fe_3_O_4_–N–C

**Figure 3:**
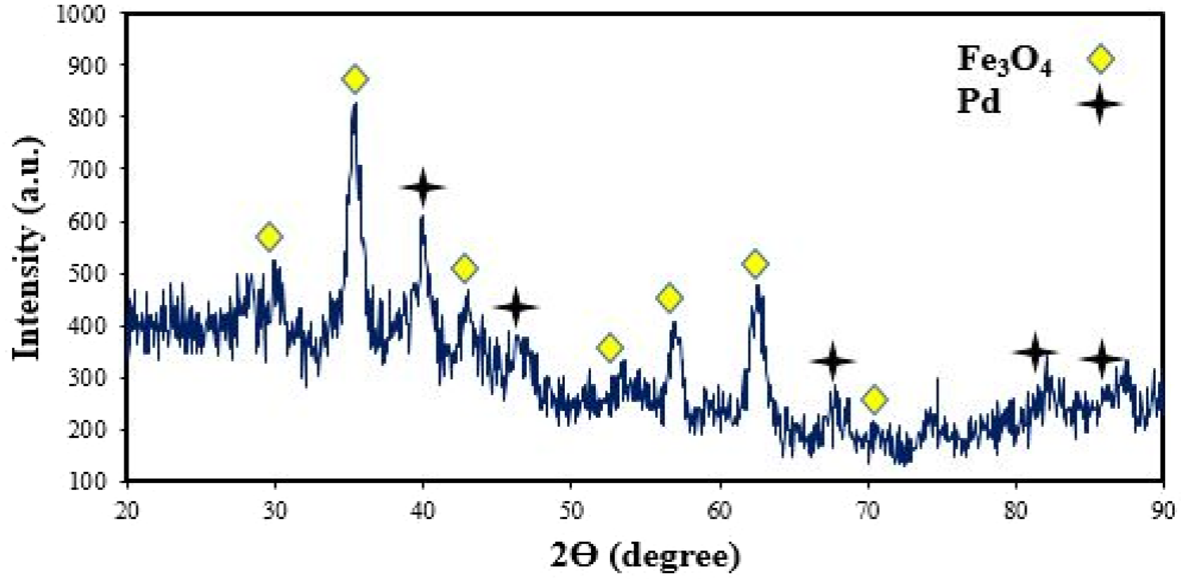
XRD pattern of Pd/Fe_3_O_4_–N–C nanocomposite.

The XRD pattern was accomplished to characterize the crystalline and phase composition of the Pd/Fe_3_O_4_–N–C nanocatalyst, illustrated in Fig. 3. The peaks at 2Ɵ = 70.9°, 62.5°, 56.92°, 53.39°, 43.04°, 35.41°, and 30.06° corresponded to the (620), (440), (511), (422), (400), (311) and (220) planes of Fe_3_O_4,_ respectively (JCPDS card no. 39–1346)^56^. The five diffraction peaks appeared at 86.12°, 81.64°, 67.76°, 46.43°, and 39.92° are respectively relevant to (222), (311), (220), (200), and (111) planes of Pd (JCPDS No. 01–087–0639)^57^.

Information obtained from the XRD pattern confirmed the constitution of Fe_3_O_4_ and Pd nanoparticles. Based on Debye-Scherer’s equation, the crystallite size of Pd and Fe_3_O_4_ nanoparticles was computed to be 29.5 nm and 17.2 nm.

#### FESEM, TEM and HRTEM

**Figure 4:**
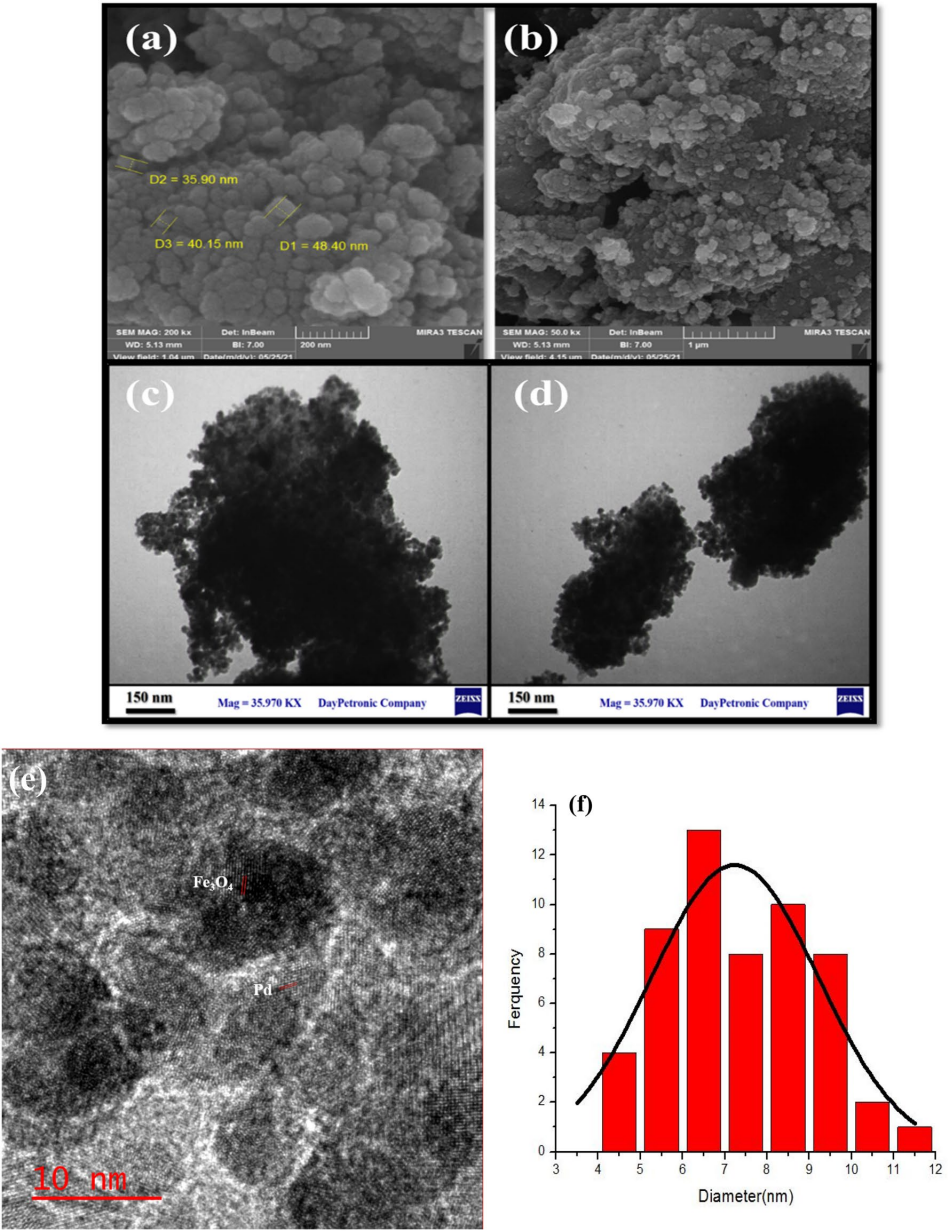
(**a**, **b**) FESEM images, (**c**, **d**) TEM images, (**e**) HRTEM, and (**f**) particle size distribution of Pd/Fe_3_O_4_–N–C nanocomposite.

SEM, TEM and HRTEM images of the Pd/Fe_3_O_4_–N–C nanocatalyst surface, as depicted in Fig. 4, were studied to assess its morphology of surface, particle size, and uniformity. Figure 4a,b reveals that Fe_3_O_4_ and Pd nanoparticles immobilized on the amorphous carbon support possess a spherical morphology with nanoscale particle size, and the estimated size of nanoparticles is 35–40 nm. These images also exhibit a well-decorated N-doped carbon substrate with nanoparticles of Pd and Fe_3_O_4_. The magnetostatic interaction between the particles led to some aggregation of Fe_3_O_4_ nanoparticles. According to the TEM images (4c, d), a good dispersion of Fe_3_O_4_ and Pd nanoparticles on the surface of Pd/Fe_3_O_4_–N–C nanocatalyst is apperceived, and the average size of the nanoparticles is 25–30 nm. Figure 4e shows an HRTEM image of a Pd/Fe_3_O_4_–N–C nanocomposite with two different planes. One of them is the (111) planes of Fe_3_O_4_ with 0.49 nm lattice spacing and the other is (111) planes of Pd with 0.23 nm lattice spacing. The particle size histogram is shown in Fig. 4f. As can be seen, the average particle size is 6–7 nm.

#### EDS-Mapping and ICP-MS

**Figure 5:**
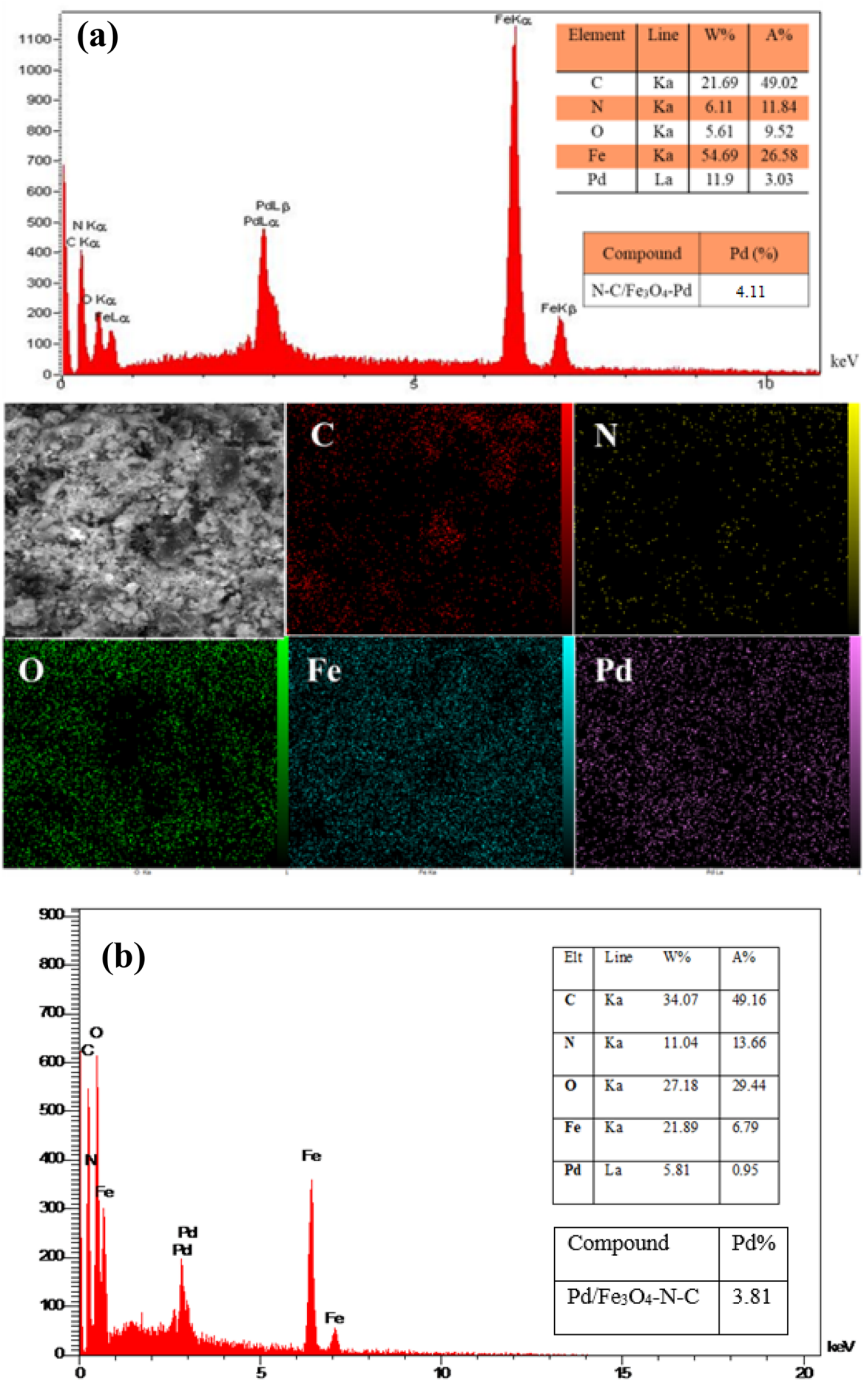

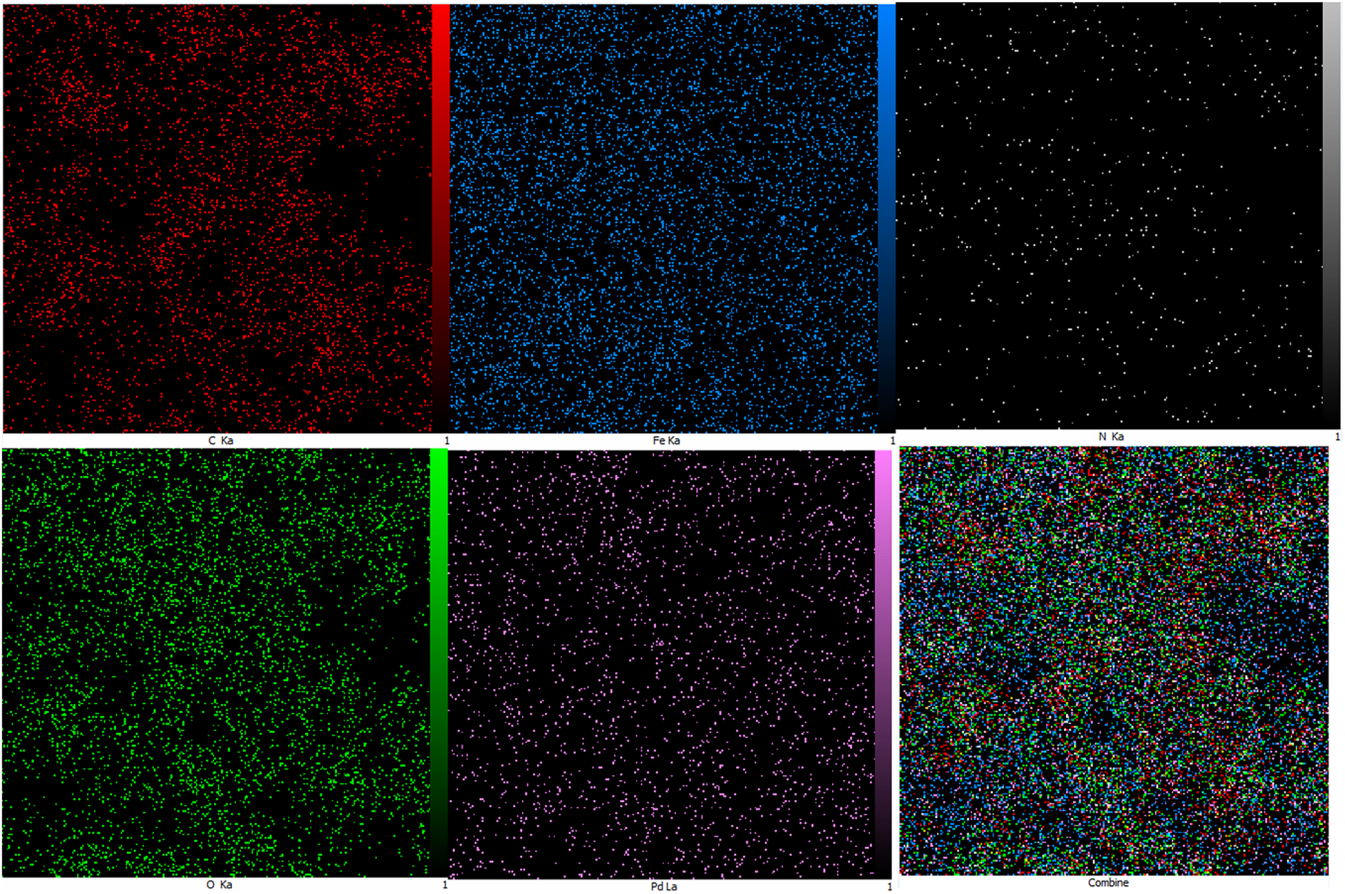
EDX-Mapping and ICP-MS analysis (**a**) fresh and (**b**) used of Pd/Fe_3_O_4_–N–C nanocomposite.

The results obtained from EDX mapping analysis in Fig. 5 confirmed the presence of carbon, nitrogen, oxygen, iron, and palladium elements in the Pd/Fe_3_O_4_–N–C nanocatalyst. Additionally, the presence of nitrogen and carbon in elemental analysis indicates that the nitrogen-doped carbon substrate is successfully composed. Thus, the synthesis of the new catalyst is confirmed wholly. Moreover, the ICP-MS test was used to prove the exact amount of Pd. This analysis illustrates that its concentration is 4.11%. To investigate the synthetic nanocatalyst stability, the used nanocatalyst by EDS-Mapping analysis and ICP was studied. The results showed that during several uses, palladium's percentage as the main center of the nanocatalyst decreased slightly (3.81%), indicating the suitable stability of the synthesized nanocatalyst.

#### N_2_ adsorption–desorption isotherm

**Figure 6:**
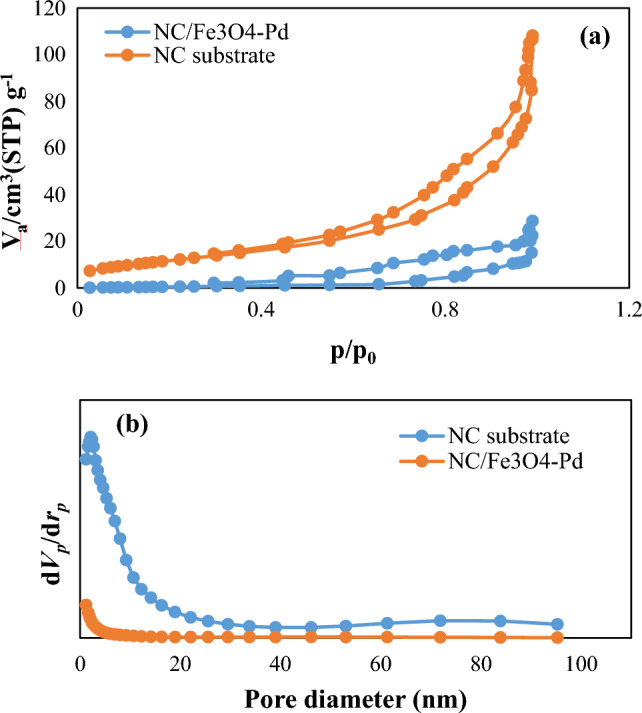
(**a**) N_2_ adsorption–desorption isotherms and (**b**) pore size distribution Of N–C substrate and Pd/Fe_3_O_4_–N–C nanocomposite.

Using the Brunauer–Emmett–Teller (BET) method, N_2_ adsorption–desorption isotherms were measured to assign the specific surface area of the nitrogen-doped carbon substrate (N–C) and Pd/Fe_3_O_4_–N–C nanocatalyst. As shown in Fig. 6a, isotherms were determined as type IV, which corresponds to the porous structure of the compounds. The BET-specific surface areas and pore volume of N–C substrate and Pd/Fe_3_O_4_–N–C nanocatalyst were computed (43.22 m^2^ g^−1^, 0.16 cm^3^ g^−1^) and (12.87 m^2^ g^−1^, 0.03 cm^3^ g^−1^), respectively. Pd/Fe_3_O_4_–N–C demonstrated a lower surface area and pore volume than the N–C substrate because some of the pores were occupied by Pd and Fe_3_O_4_ nanoparticles. Also, in Fig. 6b, the average pore size distribution of N–C substrate and Pd/Fe_3_O_4_–N–C nanocatalysts were obtained as 4.7 nm and 2.4 nm, respectively. Notably, the structure of pores is mesopores (2–50 nm). Also, the data produced from adsorption isotherms are given in Table 1.

**Table:** 

Compound	Pd%
Pd/Fe_3_O_4_–N–C	3.81

**Table 1:** BET and N_2_ adsorption/desorption isotherms data.

Sample	Surface area (m^2^ g^−1^)	Pore volume (cm^3^ g^−1^)	Average pore size (nm)
N–C substrate	43.22	0.16	4.7
Pd/Fe_3_O_4_–N–C	12.87	0.03	2.4

#### Vibration sampling magnetometer (VSM) analysis

**Figure 7:**
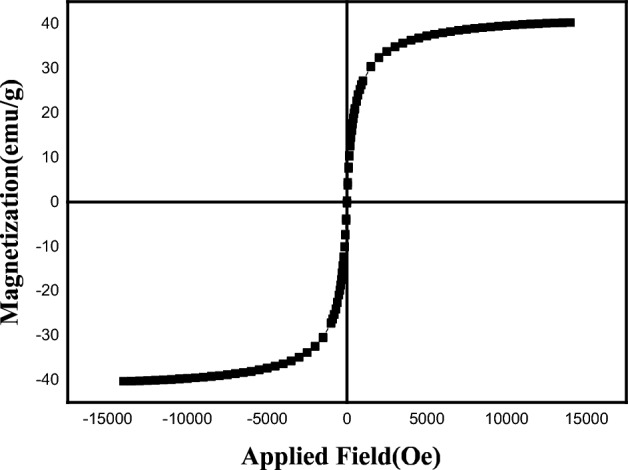
Magnetization curve of Pd/Fe_3_O_4_–N–C nanocomposite.

Utilizing the VSM technique, the magnetic feature of the Pd/Fe_3_O_4_–N–C nanocatalyst was assessed and illustrated in Fig. 7. Based on the curve, Pd/Fe_3_O_4_–N–C has magnetic properties with a saturation magnetization (M_s_) value of 40.3 emu/g. Also, this nanocatalyst exhibited superparamagnetic properties owing to its lack of a hysteresis loop. The superparamagnetic behavior of Pd/Fe_3_O_4_–N–C affords particles to collect quickly in the attendance of an outer magnetically field. Anyway, as soon as the outer field is deleted the particles are easily diffuse.

**Figure 8:**
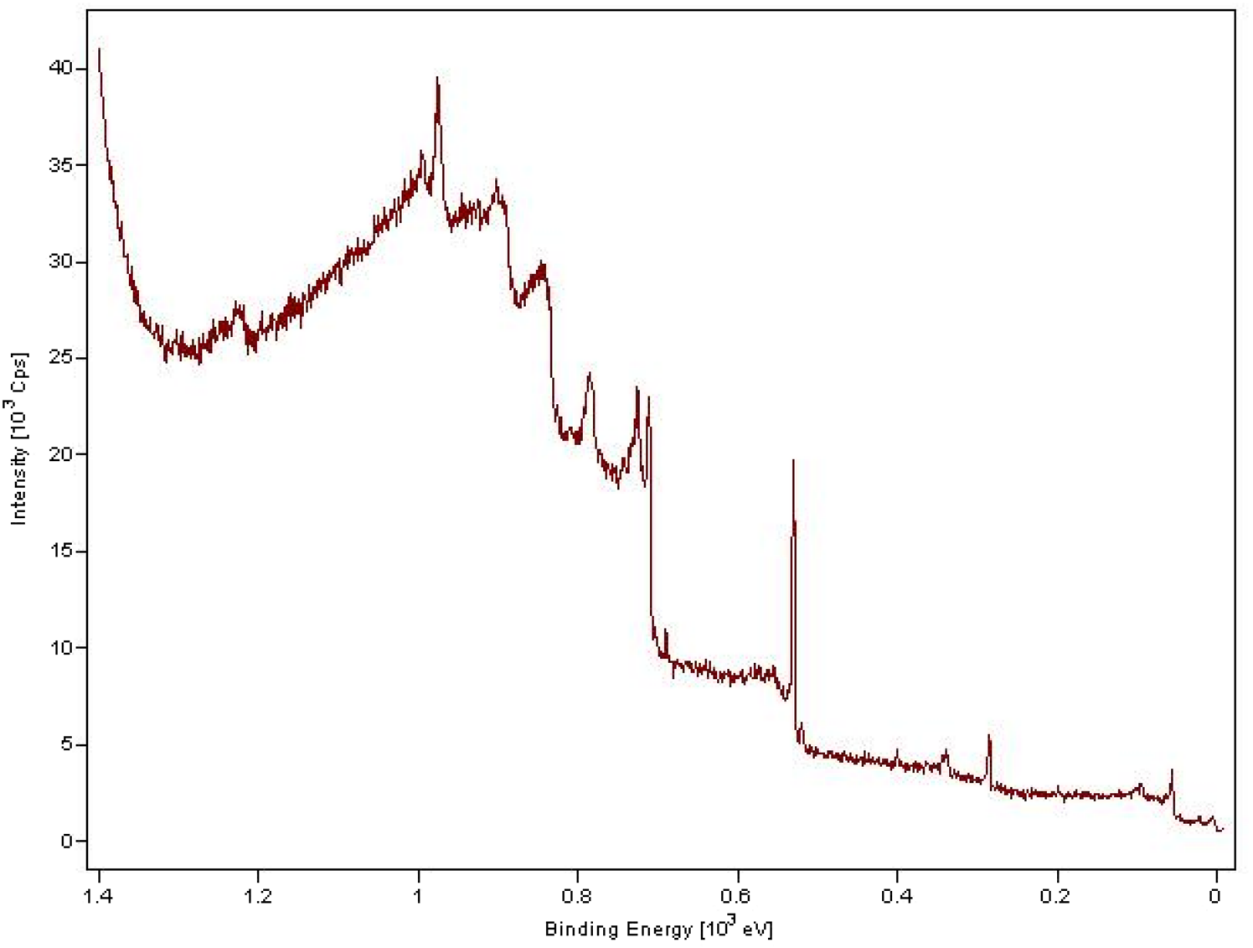
XPS of Pd/Fe_3_O_4_–N–C nanocatalyst.

An effective approach for examining the electron characteristics of the species generated on the surface is X-ray photoelectron spectroscopy (XPS), which can reveal information on the environment of the electrons, their oxidation state, and the binding energy of the metal's core electron. The Pd/Fe_3_O_4_–N–C XPS spectrum is displayed in Fig. 8. Fe 2p's XPS spectrum has two peaks in it. At 712.6 and 726.2 eV, respectively, there are two significant peaks that correspond to the usual Fe 2p1/2 and Fe 2p3/2 XPS signals of magnetite. Furthermore, the Pd nanoparticles are stable in their metallic form in the nanocomposite structure, as shown by peaks for Pd 3p3/2 and Pd 3p1/2 at 531.8 and 553.4 eV, respectively, in the Pd/Fe_3_O_4_–N–C study at the Pd 3p level. The Pd peaks in the Pd/Fe_3_O_4_–N–C shifted to lower binding energies than Pd0 standard binding energies (Pd 3p3/2 of about 532.4 eV and Pd 3p1/2 of about 560.2 eV). It has been reported that the position of the Pd 3p peak is usually influenced by the local chemical/physical environment around Pd species besides the formal oxidation state, and shifts to lower binding energy when the charge density around it increases. In the XPS elemental scan of the catalyst, the peaks for oxygen (O 2 s), carbon (C 1 s), and nitrogen (N 1 s) are also clearly discernible.

### Optimum conditions for the nitro compounds reduction reaction

As a model reaction to optimize the nitroaromatic compounds reduction conditions, the reduction of 4-nitrophenol (0.5 mmol) was assessed. Therefore, the amount of the Pd/Fe_3_O_4_–N–C nanocatalyst, type of solvent, and temperature were evaluated, as shown in Table S1. To this end, outset, nanocatalyst was discussed in various amounts. The experiments illustrated that in the absence of the catalyst, a reduction reaction did not occur, so the presence of Pd/Fe_3_O_4_–N–C nanocatalyst is necessary to perform the reaction (Table S1, Entry 1). In line with the results, 5 mg of Pd/Fe_3_O_4_–N–C was selected as the optimal amount of nanocatalyst (Table S1, Entry 2–5). Moreover, the increased amount of nanocatalyst caused an increment in yield and a shorter reaction time.

In addition, the activity of the nanocatalyst before and after the addition of Fe_3_O_4_ nanoparticles was investigated, the efficiency of the nanocatalyst did not change significantly with the addition of Fe_3_O_4_ nanoparticles, which indicates that Fe_3_O_4_ nanoparticles only facilitate the separation of the nanocatalyst from the reaction medium and have no significant effect on catalytic activity.

After determining the optimal amount of Pd/Fe_3_O_4_–N–C nanocatalyst, to peruse the effect of temperature on reaction progress, the model reaction was conducted at 25 °C and 50 °C (Table S1, Entry 6). The proper and ideal reaction temperature was 25 °C owing to the green chemistry laws and less energy expenditure.

Eventually, the model reaction was accomplished with several solvents (Table S1, Entries 7–13). As determined by the results, water represented the best performance with a 98% yield and was selected as the optimal solvent because of being environmentally friendly and inexpensive.

Following the determination of optimal conditions, to verify the effectiveness of Pd/Fe_3_O_4_-N–C nanocatalyst, the reduction reaction of various types of nitroaromatics was investigated under optimal conditions, and the results are indicated in Table 2.

**Table 2:** The reduction of nitroaromatic compounds utilizing Pd/Fe_3_O_4_–N–C.


Entry	Nitroaromatic	Product	Time (min)	Yield (%)^**b**^	Melting point /m.p.Lit (°C)^6^
1			7	> 99	186/187–190
2			10	> 99	173–174/170–174
3			35	> 98	148/145–147
4			80	90	101/102–104
5			45	97	93–95/94–98
6			40	97	105–107/103–106
7			30	> 98	76–78/77–79
8			15	97	60–64/64–66
9			25	95	Oil/-6
10			40	98	Oil
11			45	97	229/226–228

^**a**^Reaction Condition: Nitro compounds (0.5 mmol), Catalyst (5 mg), H_2_O (3 mL), NaBH_4_ (3 mmol), 25 °C.

^**b**^Isolated yield.

^**c**^EtOH/H_2_O (1:2).

### Comparison of Pd/Fe_3_O_4_–N–C catalytic activity and other catalytic systems reported in the hydrogenation of 4-nitrophenol

Catalytic performance of Pd/Fe_3_O_4_–N–C was compared to some recent catalysts, and the results were reported in Table 3. As can be seen, all catalysts illustrated admissible performance toward hydrogenation of nitroaromatics, however, Pd/Fe_3_O_4_–N–C nanocatalyst exhibited more notable activity than reported catalysts. One of the remarkable benefits of this catalyst is using glucose and histidine as bio-friendly and green precursors. This work has some benefits compared to the reported catalyst—for instance, mild reaction conditions such as green solvent, low temperature, and short reaction time.

**Table 3:** Comparison of the catalytic activity of Pd/Fe_3_O_4_–N–C and other reported catalytic systems in 4-nitroaniline hydrogenation.

Entry	Catalyst (mg)	Reaction condition	Time/h	Conv. (%) [ref]
1	Pd/ox-CEINs (50)	EtOH, HCOONH_4_, 70 °C	1	97^58^
2	SiO_2_@APTES@β-CD@Pd-PDR (20)	H_2_O, NaBH_4_, rt	3	93^59^
3	Pd@NH_2_-HMONs (10)	EtOH, H_2_, 25 °C	1	100^60^
4	Fe_3_O_4_@SiO_2_/Schiff base/Pd(II) (20)	EtOH, hydrazine hydrate, reflux	1.5	90^61^
5	RGO–Ni (10)	NaBH_4_, H_2_O, 30 °C	3:10	93^62^
6	GO–Chit–Pd (3)	NaBH_4_, H_2_O, 50 °C	20 min	92^63^
7	Pd/B-CP-HIm (30)	EtOH/H_2_O, H_2_, 50 °C	1.5	100^64^
8	Pd/CNT-P (10)	EtOH/H_2_O, H_2_, 40 °C	2	95^17^
9	Pd/Fe_3_O_4_–N–C (5)	NaBH_4_, H_2_O, r.t	35 min	> 99 [this work]

### Reusability study of the Pd/Fe_3_O_4_–N–C nanocatalyst in the hydrogenation of nitroaromatics

**Figure 9:**
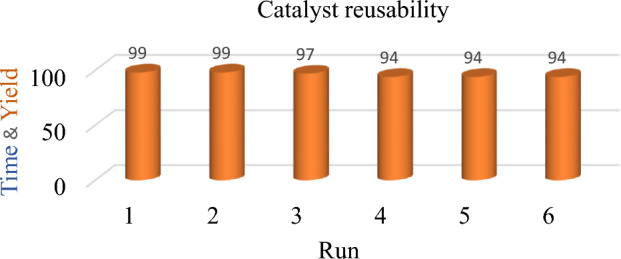
Reusability of Pd/Fe_3_O_4_–N–C nanocatalyst.

In a study on the reusability and recyclability of the Pd/Fe_3_O_4_–N–C catalyst for the reduction of nitroarenes, the catalyst displayed remarkable recyclability. A magnet was used to separate the catalyst from the reaction mixture, and then it was repeatedly cleaned in ethanol before being used in the following cycle. Figure 9 showed that catalysts may be recycled up to six times without significantly altering their weight or performance.

## Conclusion

In order to create a new reusable magnetic nanocatalyst that is N-doped porous and magnetic and immobilized by Pd nanoparticles, a straightforward and effective ionothermal approach has been developed in this research. The porous N–C substrate used to make this nanocatalyst offered a large number of active sites for the even distribution of Pd nanoparticles. The Pd/Fe_3_O_4_–N–C was effectively synthesized and employed as an effective heterogeneous nanocatalyst in reducing nitroaromatic compounds based on the results of the various characterization procedures. In the presence of a Pd/Fe_3_O_4_–N–C nanocatalyst (5 mg), 4-nitrophenol in an aqueous medium was reduced with an efficiency of > 99% over a period of 7 min. The Pd/Fe_3_O_4_–N–C nanocatalyst could be separated using an external magnet and reused up to six times without significant changes in performance. The synergetic Fe and N active sites in Pd/Fe_3_O_4_–N–C gave it a higher efficiency than other known catalysts. Because of these benefits, catalyst provision is quite valuable for real-world applications.

### Supplementary Information

Supplementary Tables.

## Data Availability

All data generated or analyzed during this study are included in this published article.
