# Supplementary material for: Ionothermal synthesis of magnetic N-doped porous carbon to immobilize Pd nanoparticles as an efficient nanocatalyst for the reduction of nitroaromatic compounds
Source: Sci Rep. 2023 Oct 16;13:17566. doi: 10.1038/s41598-023-35998-5 (PMC10579375; doi:10.1038/s41598-023-35998-5)
Supplement: Supplementary file 1 — Supplementary Tables. [file 41598_2023_35998_MOESM1_ESM.docx]

**Table S1.** The effect of Pd/Fe_3_O_4_-N-C nanocatalyst amount, Temperature, and Solvent **^a^**

|  | | | | | |
| --- | --- | --- | --- | --- | --- |
| Entry | Catalyst (mg) | T (°C) | Solvent | Time (min) | Yield (%)**^b^** |
| 1 | - | 25 | H_2_O | 180 | Trace |
| 2 | 3 | 25 | H_2_O | 15 | 94 |
| 3 | 4 | 25 | H_2_O | 10 | 95 |
| **4** | **5** | **25** | **H_2_O** | **7** | **98** |
| 5 | 6 | 25 | H_2_O | 5 | 98 |
| 6 | 5 | 50 | H_2_O | 5 | 98 |
| 7 | 5 | 25 | H_2_O: Ethanol (1:1) | 50 | 40 |
| 8 | 5 | 25 | Ethanol | 60 | Trace |
| 9 | 5 | 25 | Methanol | 60 | 20 |
| 10 | 5 | 25 | DMF | 60 | Trace |
| 11 | 5 | 25 | DMSO | 60 | Trace |
| 12 | 5 | 25 | THF | 60 | Trace |
| 13 | 5 | 25 | CHCl_3_ | 60 | Trace |
| **^a^** Reaction Condition: 4-Nitrophenol (0.5 mmol), Solvent (3 mL), NaBH_4_ (3 mmol)  **^b^** Isolated yield | | | | | |
